# Supplementary figures and images for: Extensive Changes in Transcription Dynamics Reflected on Alternative Splicing Events in Systemic Lupus Erythematosus Patients
Source: Genes (Basel). 2021 Aug 18;12(8):1260. doi: 10.3390/genes12081260 (PMC8392844; doi:10.3390/genes12081260)

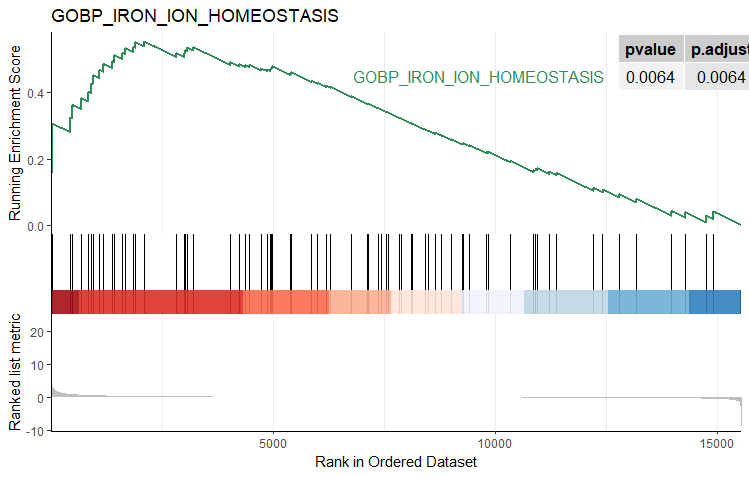

Supplement: Supplementary file 1 [file genes-12-01260-s001.zip › FinalSupplementaryFiles/FigS2-gsea_IRON_ION_HOMEOSTASIS.png]

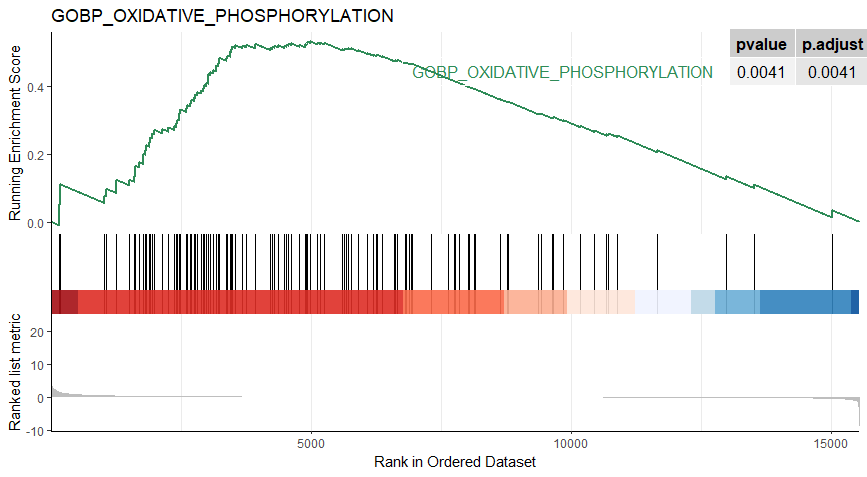

Supplement: Supplementary file 1 [file genes-12-01260-s001.zip › FinalSupplementaryFiles/FigS2-gsea_OXIDATIVE_PHOSPHORYLATION.png]

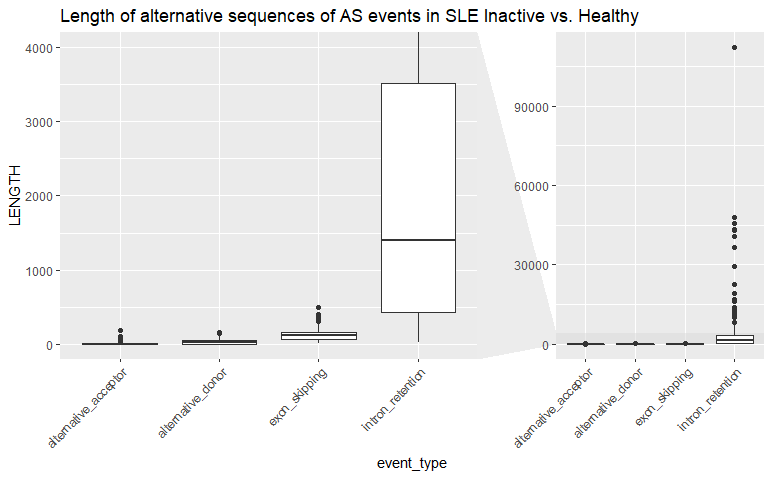

Supplement: Supplementary file 1 [file genes-12-01260-s001.zip › FinalSupplementaryFiles/FS1a.png]

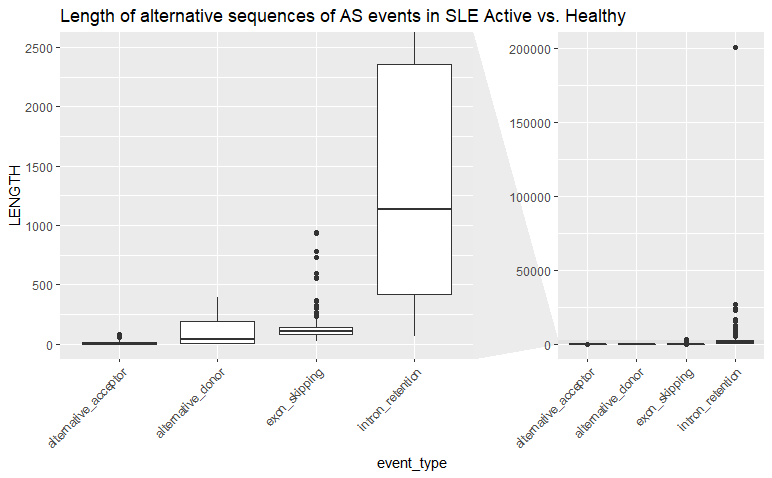

Supplement: Supplementary file 1 [file genes-12-01260-s001.zip › FinalSupplementaryFiles/FS1b.png]
